# Enhancing Long-Read-Based Strain-Aware Metagenome Assembly

**DOI:** 10.3389/fgene.2022.868280

**Published:** 2022-05-13

**Authors:** Xiao Luo, Xiongbin Kang, Alexander Schönhuth

**Affiliations:** ^1^ Genome Data Science, Faculty of Technology, Bielefeld University, Bielefeld, Germany; ^2^ Life Science and Health, Centrum Wiskunde and Informatica, Amsterdam, Netherlands

**Keywords:** long reads, haplotype, strain, metagenome, genome assembly

## Abstract

Microbial communities are usually highly diverse and often involve multiple strains from the participating species due to the rapid evolution of microorganisms. In such a complex microecosystem, different strains may show different biological functions. While reconstruction of individual genomes at the strain level is vital for accurately deciphering the composition of microbial communities, the problem has largely remained unresolved so far. Next-generation sequencing has been routinely used in metagenome assembly but there have been struggles to generate strain-specific genome sequences due to the short-read length. This explains why long-read sequencing technologies have recently provided unprecedented opportunities to carry out haplotype- or strain-resolved genome assembly. Here, we propose MetaBooster and MetaBooster-HiFi, as two pipelines for strain-aware metagenome assembly from PacBio CLR and Oxford Nanopore long-read sequencing data. Benchmarking experiments on both simulated and real sequencing data demonstrate that either the MetaBooster or the MetaBooster-HiFi pipeline drastically outperforms the state-of-the-art *de novo* metagenome assemblers, in terms of all relevant metagenome assembly criteria, involving genome fraction, contig length, and error rates.

## 1 Introduction

Microbial communities from the environment such as the sea, soil, and human gastrointestinal tract often constitute various microorganisms with uneven abundances, thus showing high diversity. The rapid variation of microorganisms may lead to multiple strains of the same species, which further elevates the biodiversity and complexity of microbial communities. Related studies have demonstrated that different strains may show different phenotypes or play different biological functions in the environment (Marx, 2016; Van Rossum et al., 2020). For example, *Escherichia coli* (*E. coli*) is usually harmless and common in the gut of mammals, but unusual strains such as *E. coli* (STEC) O26:H11 and *E. coli* (EHEC) O104:H4 are highly pathogenic and cause bloody diarrhea or even death (Burger, 2012; Bonanno et al., 2015); different strains of pathogen *Staphylococcus aureus* and *Streptococcus pyogenes* could induce significantly varying human immune responses (Sela et al., 2018); some strains of *Helicobacter pylori* (e.g., *cagA*-positive strains) are correlated with a higher risk of gastric cancer (Cover, 2016). Therefore, deciphering the strain diversity of microbial communities plays a crucial role in various applications.

In general, amplicon sequencing (e.g., 16S/18S rRNA or ITS) and traditional cultivation-based methods overlook the vast majority of genetic material, whereas metagenomic sequencing enables genome-scale assembly and strain-level analysis. Strain-resolved *de novo* genome assembly is the ultimate solution to decipher strain diversity in metagenomic samples; however, this problem has largely remained unresolved so far. Notably, here we define the concept of “strain” to agree with that of a “haplotype”, which is a non-redundant genome sequence with sufficient sequencing coverage. This is in accordance with the definition of a strain in the related work (Vicedomini et al., 2021).

Next-generation sequencing (NGS) has been routinely used in *de novo* metagenome assembly, and many related tools have been developed such as SPAdes (Bankevich et al., 2012), IDBA-UD (Peng et al., 2012), and MEGAHIT (Li et al., 2015). These tools only generate squashed assemblies at the species level without considering strain diversity. In addition, VG-flow (Baaijens et al., 2020) performs strain-aware metagenome assembly at the whole genome scale, whereas Snowball (Gregor et al., 2016) and DESMAN (Quince et al., 2017) do so only at the gene scale. These *de novo* assembly methods struggle to generate long-strain-specific genome sequences because NGS reads are too short (hundreds of bp) to span inter-/intra-genomic repeats.

Third-generation sequencing (TGS) technologies such as Pacific Biosciences (PacBio) and Oxford Nanopore Technologies (ONT) have revolutionized the method of DNA sequencing because they generate considerably longer reads (tens of Kbps to Mbp). Hence, they have recently provided unprecedented opportunities to carry out haplotype- or strain-resolved *de novo* genome assembly. In particular, PacBio high-fidelity (HiFi) long reads are highly accurate (sequencing error rate <1% ) such that haplotype- or strain-resolved genome assembly can be achieved relatively easily. Related works such as HiCanu (Nurk et al., 2020), hifiasm (Cheng et al., 2021), and hifiasm-meta (Feng et al., 2021) are designed to assemble high-fidelity long reads without losing haplotype information. Nevertheless, other types of long reads (i.e., PacBio CLR, ONT) usually suffer from high sequencing error rate (5–15%). This makes it more difficult to perform strain-resolved metagenome assembly mainly due to the hindrance in distinguishing low frequent strain variations from sequencing errors. Related approaches such as Canu (Koren et al., 2017) and metaFlye (Kolmogorov et al., 2020) as the most dominant metagenome assemblers are unable to separate strains of divergence vary by less than 5%; note that the majority of different strains from one species vary by less than 5%. Later, Strainberry (Vicedomini et al., 2021) was proposed as a post-hoc strain resolver based on pre-assembled contigs from Canu or metaFlye. However, it is only suitable to deal with low-complexity samples and the final result largely depends on the performance of the pre-assemblers used. Overall, there is plenty of room to improve the strain-aware metagenome assembly from error-prone long reads. In addition, previous studies (Luo et al., 2021; Luo et al., 2022) have demonstrated that performing haplotype-aware error correction in noisy long reads prior to genome assembly can significantly improve haplotype awareness.

Here, we propose MetaBooster and MetaBooster-HiFi, as two pipelines for strain-aware metagenome assembly from PacBio CLR and Oxford Nanopore long-read sequencing data. First, MetaBooster/MetaBooster-HiFi performs error correction on raw reads using VeChat (Luo et al., 2022), a new variation graph-based standalone tool to correct errors in long reads. Subsequently, reads corrected by VeChat are combined with reads corrected by Canu, a state-of-the-art long-read assembler (Koren et al., 2017). A crucial insight is that joining the two read sets, and thereby joining the (obviously complementary) virtues of VeChat and Canu creates maximum synergy, which considerably “boosts” the performance of assemblers that one subsequently uses. Here, we focus on assembling the joined reads using either Canu itself (*MetaBooster*), or the PacBio HiFi mode of Canu (*MetaBooster-HiFi*).

In this study, we do not present a new method per se; however, we do present a pipeline that is superior over all conceivable *de novo* long-read-based metagenome assembly strategies presented so far, by fairly large margins. In other words, the primary novelty of this work is the superior performance of the “boosted” error correction—assembly pipeline. An additional novelty is the introduction of VeChat, as a variation graph-based error correction tool for long reads into the world of *de novo* metagenome assembly.

Benchmarking experiments on both simulated and real sequencing data demonstrate that either the MetaBooster or the MetaBooster-HiFi pipeline drastically outperforms the existing *de novo* metagenome assemblers in terms of relevant assembly evaluation criteria such as genome fraction, contig length, and error rate. Of further interest, applying Strainberry as a post-hoc tool for revisiting the resulting assemblies, improves the assemblies of MetaBooster and MetaBooster-HiFi by further small amounts. In comparison with the strategies originally suggested in Strainberry, one achieves further increases in performance.

## 2 Materials and Methods

### 2.1 Workflow

**FIGURE 1:**
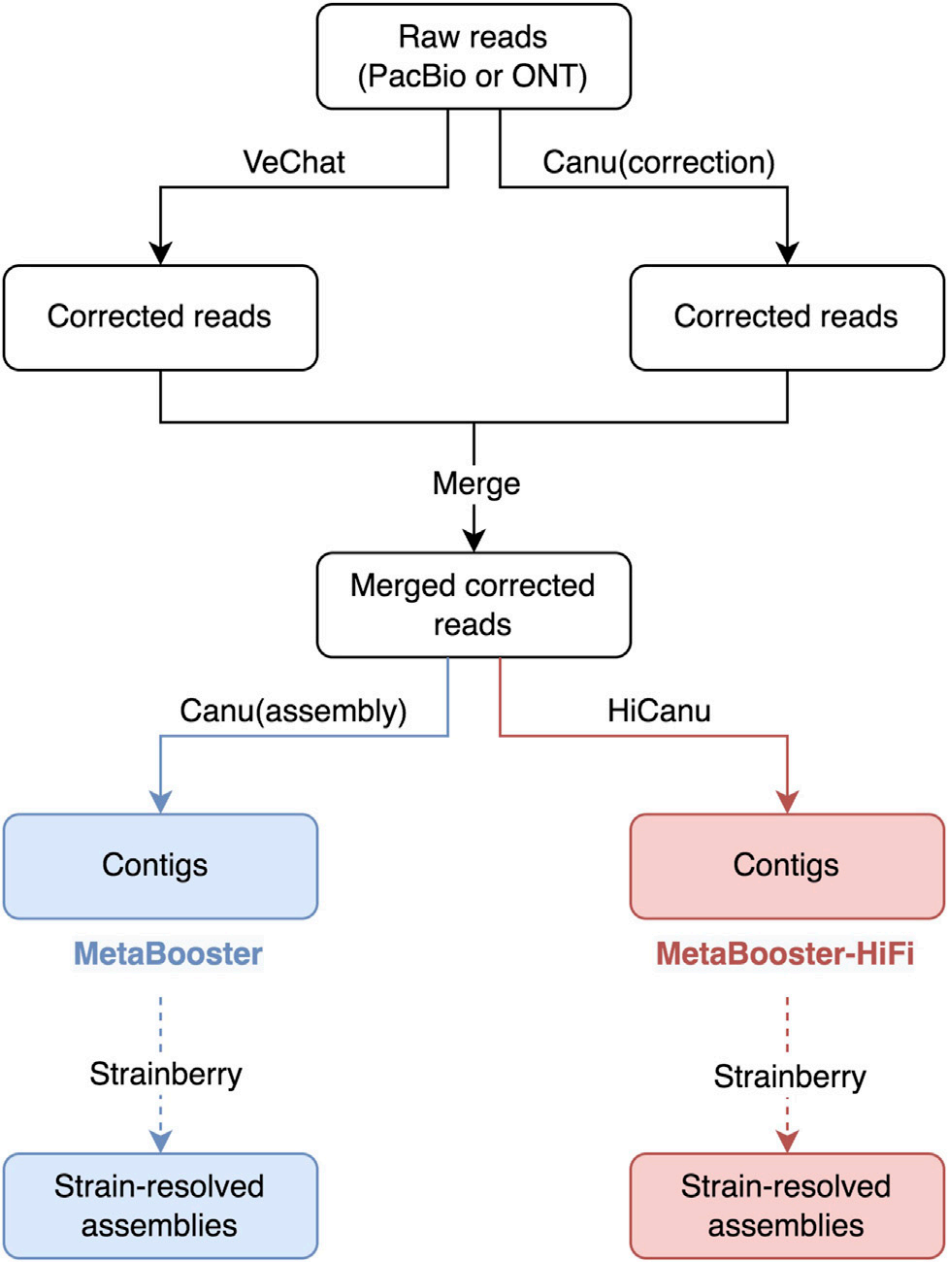
Workflow overview of MetaBooster and MetaBooster-HiFi. MetaBooster takes raw reads as input and outputs contigs using Canu (blue), whereas MetaBooster-HiFi takes raw reads as input and outputs contigs using HiCanu (red). The dotted arrow lines denote running Strainberry is optional. Canu (correction) means running Canu’s correction and trimming modules only. Canu (assembly) means running Canu’s assembly module only.

See Figure 1 for an overview of the workflow of the two pipelines, MetaBooster and MetaBooster-HiFi. Firstly, raw reads are corrected using VeChat (Luo et al., 2022), on the one hand (left branch at the top in Figure 1) and the error correction and read trimming module of Canu (Koren et al., 2017), on the other hand (right branch at the top in Figure 1). In detail, we use the reads, as provided through the file $prefix.trimmedReads.fasta, as a standard output that is generated by Canu, and reflects the corrected reads that Canu uses for the assembly. Subsequently, corrected reads from VeChat and Canu are merged. This doubles the number of original reads on the one hand, without merely duplicating them. Reads that are not well corrected by one of the methods can be corrected by the other method, because error correction modules work according to different principles. The resulting joined reads can then be assembled using Canu’s assembly module (the blue branch, named MetaBooster, at the bottom of Figure 1) or HiCanu (Nurk et al., 2020) (the red branch, named MetaBooster-HiFi, at the bottom of Figure 1), respectively.

Eventually, as an optional last step, one can apply Strainberry to post-process assemblies and further improve them; see the dotted lines in Figure 1.

### 2.2 Datasets

To evaluate the two new pipelines, with or without the additional application of Strainberry, in comparison with all state-of-the-art *de novo* strain-aware metagenome assembly protocols that one can currently conceive, we make use of simulated and real data. While the simulated data is generated using well-established simulators, we draw real data from recent publications on the topic. See Supplementary Table S1 for the details of all data sets, such as names and accession IDs of species, number of strains per species, and genome divergences.

#### 2.2.1 Simulated Sequencing Data

We used CAMISIM (Fritz et al., 2019) to simulate metagenomic data (PacBio CLR reads) and PBSIM2 (Ono et al., 2021) as a read simulator instead of the built-in one in CAMISIM. For PBSIM2, the built-in P6C4 model-based simulation profiles were used. These reflect the features of error-prone long reads generated by the currently most popular sequencing platforms. To evaluate the effects of varying numbers of strains and divergence, as well as sequencing coverage when running our pipelines and the other methods, we simulated both a low-complexity data set that contains a smaller amount of strains and a high-complexity data set that is characterized by containing considerable more strains.

In detail, the low-complexity data set consists of seven species, together presenting 13 strains, at an average nucleotide identity (ANI) of <98.4% . All ANI values were computed using FastANI (an alignment-free method to estimate ANI) with default parameters (Jain et al., 2018). The high-complexity data set consists of 30 species that together give rise to 100 strains in total, at an ANI of <99.99% . For both data sets, the average sequencing coverage of strains was approximately 30x. The average sequencing error rate was 10%. The relative abundance of strains varied from 3.1 to 19.8% for the low-complexity data and from 0.28 to 3.3% for the high-complexity data.

The genomes serving as a template for both simulated data sets were retrieved from (Quince et al., 2017); note that these genomes were already used in several studies before (Nicholls et al., 2021; Luo et al., 2022).

#### 2.2.2 Real Sequencing Data

Mock community I: natural whey starter culture data. We downloaded two real metagenomic datasets (PacBio CLR reads) derived from natural whey starter cultures (NWCs), as presented by Somerville et al., 2019. We then merged the two data sets. We further subsampled 20% of the reads from the merged data set, which yielded a real metagenomic data set of relatively low complexity, dominantly containing three species together exhibiting six strains (each species involves two strains). The ANI between the two strains (from the same species) ranges from 98.03 to 99.99%. The average sequencing coverage is about 50x. Note that this data comes from real samples, thus it might contain more than the six strains (Somerville et al., 2019).We use these six dominant strains as reference genomes for assembly evaluation because it is the only viable option.

Mock community II: Microbial 10-plex data. We downloaded raw long-read sequencing data and the corresponding reference genomes referring to a 10-plex (as multiplexed) dataset that was sequenced using the PacBio Sequel System, Chemistry v3.0 (https://downloads.pacbcloud.com/public/dataset/microbial_multiplex_dataset_release_SMRT_Link_v6.0.0_with_Express_2.0/). Subsequently, we randomly subsampled 10% of the reads, which yielded a metagenome data set reflecting a mock community of an average sequencing coverage of approximately 40x. The data set contains seven species showing nine strains overall, reflecting an ANI of <98.5% as an indicator for the divergence of strains.

Real human gut microbiome data: We downloaded ONT reads reflecting a real human gut sample that can be accessed at SRR8427258 and was presented and analyzed earlier (Moss et al., 2020). We also obtained Illumina reads of the same sample (accessible through identifiers SRR6807561, SRR6788327) as provided through another study (Bishara et al., 2018). The Illumina reads were used to evaluate assembly performance in a reference-free manner.

### 2.3 Assembly Evaluation

The metagenome assembly performance was evaluated by means of several commonly used metrics, routinely reported by MetaQUAST V5.1.0rc1 (Mikheenko et al., 2018), as a prominent assembly evaluation tool. See below for specific explanations (and see http://quast.sourceforge.net/docs/manual.html for full details). Contigs with lengths less than 500bp were filtered before evaluation, reflecting common practice. In particular, we ran the metaQUAST.py program with the option “--unique-mapping” appropriately taking into account that our data sets reflect mixed samples. In addition, for the purpose of evaluating the real human gut sample, there is no ground truth in the reference genomes. Hence, Merqury (Rhie et al., 2020) was used to evaluate results based on auxiliary short-read sequencing data, in a reference-independent manner.

#### 2.3.1 Genome Fraction

The Genome fraction is the percentage of aligned bases in the ground truth strains covered by output contigs. This measures how complete the reference genomes are covered by contigs. When reference genomes are unknown, k-mer completeness reported from Merqury is used as an alternative metric of approved usefulness (Rhie et al., 2021; Luo et al., 2021).

#### 2.3.2 N50 and NGA50

N50 is defined as the length for which the collection of all contigs of that length or longer covers at least half the assembly. NGA50 is similar to N50 but can only be calculated when the reference genome is provided. NGA50 only considers the aligned blocks (after breaking reads/contigs at misassembly events and trimming all unaligned nucleotides). NGA50 is then defined as the length for which the overall size of all aligned blocks of this length or longer equals at least half of the reference haplotypes. Both N50 and NGA50 are used to measure the contiguity of the assemblies.

#### 2.3.3 Error Rate (ER)

The error rate is equal to the sum of the mismatch and the indel rate when mapping the obtained contigs to the reference haplotype sequences.

#### 2.3.4 N-Rate

The N-rate is defined as the proportion of ambiguous bases (‘N’s) in the assembly.

#### 2.3.5 Number of Misassemblies (#Misassemblies)

The misassembly event in assemblies indicates that left and right flanking sequences align to the true haplotypes with a gap or overlap of more than 1 kbp, or align to different strands, or even to different strains. Here, we report the total number of misassemblies in the given sequence data.

#### 2.3.6 Duplication Ratio

The duplication ratio is equal to the total amount of aligned bases in the assembly divided by the total amount of aligned bases in the reference sequences.

## 3 Results

### 3.1 Simulated Data

See Table 1 for the assembly performance of simulated sequencing datasets. Both MetaBooster-HiFi and MetaBooster generate better assemblies in terms of various aspects, in comparison to readily available assemblers (i.e., Canu and metaFlye). For example, on the low-complexity dataset, MetaBooster-HiFi achieves a 7.1% higher genome fraction, about 10 times longer NGA50 while maintaining fewer misassemblies, and 3.6 times lower error rate, compared with those of Canu (better assemblies than metaFlye). Likewise, on the high complexity dataset, MetaBooster-HiFi achieves 9.3% higher genome fraction, about two times longer NGA50 while maintaining fewer misassemblies, and 1.2 times lower error rate, compared with those of Canu (better assemblies than metaFlye). Moreover, when making additional use of the post-hoc strain resolver, Strainberry, one receives assemblies that are further improved in terms of genome fraction and error rate, however, at the cost of contiguity (N50/NGA50). In comparison with combining other assemblers with Strainberry, MetaBooster/MetaBooster-HiFi + Strainberry still outperform the other combinations (i.e., Canu/metaFlye + Strainberry), especially in terms of genome fraction, N50/NGA50, and error rate. Note that MetaBooster/MetaBooster-HiFi + Strainberry achieve only small absolute improvements over the other combinations (i.e., Canu/metaFlye + Strainberry) on simulated data. However, from a relative perspective, MetaBooster/MetaBooster-HiFi + Strainberry approximately reduces half of the error rate and the amount of misassemblies on low complexity data, and MetaBooster-HiFi nearly doubles the NGA50 on the high complexity data. Note also that metaFlye + Strainberry has a high ambiguous base rate (N (%)), comparable with its error rate, whereas MetaBooster/MetaBooster-HiFi + Strainberry do not exhibit such effects.

**TABLE 1:** Assembly performance for simulated sequencing data. Results of assemblers are sorted by the second and the forth columns. The reference size of low complexity and high complexity data is about 63.3 Mbp and 373.9 Mbp, respectively. We set the parameter *genomeSize* as the reference size presented here when running Canu.

assembler	Post-hoc strain resolver	#Contigs	Genome fraction (%)	N50 (bp)	NGA50 (bp)	Error rate (%)	N (%)	# Misassemblies	Duplication ratio	Assembly size (Mbp)
*Low complexity*										
MetaBooster-HiFi	-	207	98.2	5,401,308	3,809,573	0.041	0.000	13	1.04	65.0
MetaBooster	-	229	93.5	1,776,642	1,191,789	0.059	0.000	41	1.03	61.8
Canu	-	336	91.1	670,907	382,472	0.149	0.000	65	1.05	61.5
metaFlye	-	660	71.6	212,939	74,586	0.330	0.000	273	1.04	48.3
MetaBooster	Strainberry	902	99.6	791,215	1,046,223	0.035	0.000	17	1.06	66.9
MetaBooster-HiFi	Strainberry	307	99.5	1,893,577	1,907,959	0.044	0.000	15	1.05	65.9
Canu	Strainberry	879	99.4	218,802	262,285	0.086	0.000	32	1.08	68.0
metaFlye	Strainberry	894	96.5	490,469	455,481	0.094	0.068	80	1.06	64.9
*High complexity*										
MetaBooster-HiFi	-	3,734	88.8	354,665	254,136	0.162	0.000	362	1.17	394.7
MetaBooster	-	3,064	81.2	383,218	156,134	0.174	0.000	466	1.10	337.5
Canu	-	3,204	79.5	442,268	137,979	0.194	0.000	408	1.08	328.0
metaFlye	-	3,554	52.0	264,868	15,820	0.409	0.000	1,233	1.03	207.0
MetaBooster	Strainberry	9,876	93.3	87,675	98,024	0.117	0.007	236	1.17	406.8
MetaBooster-HiFi	Strainberry	6,027	93.0	133,704	155,125	0.150	0.007	209	1.19	412.9
Canu	Strainberry	10,229	92.5	80,214	87,947	0.136	0.012	213	1.16	402.8
metaFlye	Strainberry	6,211	84.8	219,010	144,318	0.136	0.182	828	1.07	343.3

### 3.2 Real Data

See Table 2 for the assembly performance referring to the real sequencing data sets, including the mock communities. On the natural whey starter culture (NWC) dataset, MetaBooster achieves the highest genome fraction (4 and 25.6% higher than that of Canu and metaFlye, respectively), comparable NGA50 while the N50 is shorter. MetaBooster also has a lower error rate compared with that of Canu, and better assemblies than that of metaFlye. Although MetaBooster-HiFi shows worse assembly performance in terms of genome fraction and N50/NGA50 compared with that of Canu, it still substantially outperforms metaFlye in terms of all key measurements.

**TABLE 2:** Assembly performance for real sequencing data. Results of assemblers are sorted by the second and the fourth columns. Note that in the ‘Real human gut microbiome’ dataset, genome fraction reflects the k-mer completeness reported by Merqury. The reference size of Mock community I and II is about 13.0 Mbp and 36.8 Mbp, respectively. The reference size of the real human gut microbiome dataset is unknown, but metaFlye generates the assembly with the size of about 192 Mbp, thus we assume the estimated reference size is 200 Mbp. We set the parameter *genomeSize* as the reference size presented here when running Canu.

assembler	Post-hoc strain resolver	#Contigs	Genome fraction (%)	N50 (bp)	NGA50 (bp)	Error rate (%)	N (%)	# Misassemblies	Duplication ratio	Assembly size (Mbp)
*Mock community I: natural whey starter culture*										
MetaBooster	-	381	88.6	93,125	137,737	0.119	0.000	376	1.51	17.5
Canu	-	191	84.6	268,047	138,616	0.132	0.000	255	1.27	14.1
MetaBooster-HiFi	-	217	79.2	159,827	88,396	0.090	0.000	131	1.23	12.9
metaFlye	-	376	63.0	109,472	22,888	0.269	0.000	381	1.17	10.0
MetaBooster	Strainberry	452	89.2	78,953	119,193	0.157	0.033	393	1.53	17.9
Canu	Strainberry	265	85.2	135,194	125,977	0.163	0.026	269	1.29	14.5
MetaBooster-HiFi	Strainberry	309	80.8	93,739	80,481	0.131	0.055	168	1.26	13.5
metaFlye	Strainberry	430	72.4	130,897	44,965	0.258	0.274	371	1.22	11.9
*Mock community II: Microbial 10-plex*										
MetaBooster-HiFi	-	1,013	91.6	180,008	347,693	0.104	0.000	90	1.29	43.8
MetaBooster	-	684	90.1	245,260	137,384	0.171	0.000	99	1.18	39.3
Canu	-	485	88.8	411,086	135,250	0.199	0.000	94	1.10	36.3
metaFlye	-	291	70.4	1,248,427	102,535	0.191	0.000	147	1.02	26.8
MetaBooster	Strainberry	1770	98.8	49,458	77,254	0.104	0.010	64	1.25	45.3
Canu	Strainberry	1,597	98.7	56,315	76,684	0.114	0.014	60	1.19	43.3
metaFlye	Strainberry	765	96.5	737,281	479,290	0.075	0.073	49	1.06	37.8
MetaBooster-HiFi	Strainberry	1,516	95.8	67,377	153,561	0.114	0.003	70	1.32	46.5
*Real human gut microbiome*										
metaFlye	-	2,990	84.3	203,880	-	2.945	0.000	-	-	191.9
MetaBooster	-	6,425	84.2	109,293	-	1.433	0.000	-	-	199.2
Canu	-	3,622	81.3	192,144	-	2.249	0.000	-	-	159.8
MetaBooster-HiFi	-	4,544	42.7	14,411	-	0.990	0.000	-	-	46.8
metaFlye	Strainberry	7,125	77.7	63,200	-	3.551	0.012	-	-	214.8
MetaBooster	Strainberry	11,012	77.4	41,569	-	2.618	0.001	-	-	222.3
Canu	Strainberry	7,996	75.7	58,104	-	3.096	0.003	-	-	181.8
MetaBooster-HiFi	Strainberry	6,830	52.5	16,534	-	1.347	1.641	-	-	59.5

When adding Strainberry, MetaBooster + Strainberry yields the highest genome fraction (89.2%) on NWC, which slightly improves on running MetaBooster alone (88.6%). This comes at the expense of shorter N50/NGA50, higher error rate, and N rate. In comparison, Canu + Strainberry achieves 85.2%, which is 4% less than MetaBooster + Strainberry.

Evaluating results on the Microbial 10-plex dataset, both MetaBooster and MetaBooster-HiFi outperform Canu and metaFlye in terms of genome fraction, NGA50, and error rate. In addition, MetaBooster + Strainberry generates better assemblies in terms of genome fraction and “error rate + N rate”, compared with other combinations of assemblers plus Strainberry. It is remarkable that the microbial 10-plex dataset is the one dataset where adding in Strainberry leads to the most pronounced improvements in terms of genome fraction, which reflects the coverage of individual strains.

On the real human gut microbiome dataset, MetaBooster achieves comparable genome fraction, 2 times shorter N50, but in compensation 2 times lower error rate in a comparison with metaFlye. Neither Canu nor MetaBooster-HiFi can rival the results of any of MetaBooster or metaFlye.

Unlike on other datasets, the integration of Strainberry worsens results in terms of coverage of strains (Genome Fraction) and error rate, where the trends to the worse are quite obvious. For example, Genome Fraction goes down from 84.3% without Strainberry to 77.4% with Strainberry, when regarding MetaBooster. This trend applies also to all other tools apart from MetaBooster-HiFi.

MetaBooster-HiFi shows bad assembly performance on this dataset. The most likely explanation is that the sequencing error rate of raw reads is so high that HiCanu fails to handle the corrected reads because the remaining error rate is too high.

*In summary,* MetaBooster and MetaBooster-HiFi introduce advantages of various, non-negligible kinds on the different real data sets: on NWC, MetaBooster demonstrates a significantly greater coverage of strains, with or without a subsequent application of Strainberry.

On Microbial 10-plex, MetaBooster is better than Canu (as the toughest rival) with or without a subsequent application of Strainberry. We observe that Microbial 10-plex is the only real dataset, where the application of MetaBooster or not remains a matter of taste, as it is reasonable to say that it shares merits with Canu.

On the real human gut microbiome data set, we have a picture that substantially differs from the other two data sets. Here, a subsequent application of Strainberry does not improve the assembly performance probably because Strainberry was developed for low-complexity metagenomes (so it is not used according to its primary purpose). When running methods/pipelines without (the rather worsening) Strainberry, MetaBooster achieves comes in second in terms of Genome Fraction, while clearly rivaling the optimal metaFlye (metaFlye: 84.3%, MetaBooster: 84.2%). In any case, MetaBooster more than halves the error rate of metaFlye.

Overall, although not dominating other approaches in every aspect, MetaBooster and MetaBooster-HiFi outperform the state-of-the-art approaches in many important aspects and therefore add important qualities that had not been available before.

### 3.3 Running Time and Memory Usage

See Supplementary Table S2 and Supplementary Table S3 for running time and memory usage statistics about runs on simulated and real sequencing data. In general, the error correction step (VeChat) is the most time-consuming step and makes for peak memory usage.

MetaBooster is 6 ∼ 66 or 6 ∼ 213 slower and requires several times higher or comparable peak memory usage than others (metaFlye and Canu) on simulated and real sequencing data, respectively. Additionally, MetaBooster-HiFi is faster than MetaBooster while needing the same peak memory usage.

## 4 Discussion

We presented MetaBooster and MetaBooster-HiFi as two novel error correction and assembly pipelines by which to perform *de novo* strain aware metagenome assembly. In experiments, we observed that MetaBooster and MetaBooster-HiFi introduce novel qualities in that respect, in terms of genome fraction (strain-specific genome coverage), error rates or contiguity of assemblies.

MetaBooster and MetaBooster-HiFi combine the error-corrected reads from VeChat, which is based on a variation graph-based method, and the ones one can retrieve from Canu’s error correction module, as an overlap graph-based method. This way, our pipelines create synergy by exploiting the advantages of the two strategies, on the one hand. To be specific, VeChat generally achieves better error correction for most reads than that of Canu as documented by Luo et al., 2022. However, VeChat may not perform very well in some particular regions, for example in highly divergent genomic regions where different strains substantially vary (see Supplementary Figure S1). Canu may achieve better error correction in such highly variable regions, because it uses a consensus-based strategy for correction. From this point of view, Canu complements the generally superior, but in difficult regions incomplete reads of VeChat wherever necessary. This shows that the joined corrected reads from Vechat and Canu can generate better assembly (see bottom two panels in Supplementary Figure S1). On the other hand, the combination itself increases the sequencing coverage. Both of these points lead to enhanced strain-aware metagenome assemblies (see Supplementary Table S4 for additional arguments about merging error correction modules).

Evaluating benchmarking results in detail indicates that in most cases, either MetaBooster or MetaBooster-HiFi pipeline outperforms Canu and metaFlye, as the state-of-the-art strain-aware *de novo* metagenome assembly tools on most datasets, in one or more of the most relevant categories, such as genome fraction (coverage of individual strains), contig contiguity and error rate. As a general trend, MetaBooster-HiFi appears to outperform MetaBooster on higher-quality data such as PacBio CLR data. MetaBooster, on the contrary, shows better performance on lower-quality data such as ONT reads. Note that the evolution of nanopore sequencing technologies is fast, with the most recently published platform being able to generate (long) reads of error rates as low as <5% . This motivates the parallel development of the MetaBooster (for today’s low-quality reads) and MetaBooster-HiFi (future long reads of higher quality), providing flexibility relative to future progress in sequencing technology. In rather unclear situations, a guideline for real practice is to run both pipelines and opt for the better one relative to the practically relevant criteria one can determine.

We also evaluated Strainberry as a post-hoc strain resolver that has the potential to enhance assemblies further. Clearly, Strainberry’s performance depends on the assemblies that one feeds into it, which are generated by independent software, where Canu and metaFlye were suggested by the developers of Strainberry. Using MetaBooster or MetaBooster-HiFi as the assembler whose output is used for Strainberry, one can achieve further improvements over the assembly strategies originally suggested by Strainberry.

Overall, while exhibiting various favorable features, a conclusive insight into the why and how of how advantages (and on rare occasions also disadvantages) is achieved by the “booster” pipelines has not yet been reached. In future work, we are planning to study how to reach optimal synergy when crafting strain aware metagenome assembly pipelines from approaches presented in the literature.

## Data Availability

Raw sequencing data and resulting assemblies can be downloaded from Zenodo (DOI: \href{https://zenodo.org/record/5830706#.YdssXWiZOw4}{https://doi.org/10.5281/zenodo.5830706}]. The source code is publicly available at \url{https://github.com/HaploKit/metagenome-asm}. Further inquiries can be directed to the corresponding author.
