# Supplementary material for: Enhancing Long-Read-Based Strain-Aware Metagenome Assembly
Source: Front Genet. 2022 May 13;13:868280. doi: 10.3389/fgene.2022.868280 (PMC9136235; doi:10.3389/fgene.2022.868280)
Supplement: Supplementary file 1 [file DataSheet1.PDF]

Supplementary Material

1 SUPPLEMENTARY TABLES AND FIGURES

1.1 Tables

Table S1. Description of simulated and real sequencing data sets. See the excel file: *Supplementary Table S1.xlsx*

	CPU time (h)	peak memory usage (GB)
Low complexity		
MetaBooster	411.6	81.2
MetaBooster-HiFi	330.6	81.2
Canu	69.5	10.8
metaFlye	10.1	20.4
High complexity		
MetaBooster	7066.8	75.5
MetaBooster-HiFi	5331.3	75.5
Canu	547.0	11.8
metaFlye	107.6	75.6

Table S2. Runtime and memory usage for metagenome assembly of simulated sequencing data.

	CPU time (h)	peak memory usage (GB)
NWC data		
MetaBooster	661.3	69.9
MetaBooster-HiFi	593.9	69.9
Canu	41.1	5.0
metaFlye	6.9	18.4
Microbial 10-plex		
MetaBooster	389.2	33.2
MetaBooster-HiFi	335.4	33.2
Canu	63.0	5.2
metaFlye	7.9	19.5
Real human gut microbiome		
MetaBooster	12224.8	31.2
MetaBooster-HiFi	7812.6	22.6
Canu	2143.4	10.8
metaFlye	57.5	61.4

Table S3. Runtime and memory usage for metagenome assembly of real sequencing data.

1.2 Figures

Error corrector	Assembler	#Contigs	Genome fraction (%)	N50 (bp)	NGA50 (bp)	Error rate (%)	N(%)	# Misass-emblies	Duplication ratio	Assembly size(Mbp)
-	MetaBooster-HiFi	217	98.2	3809619	3809619	0.037	0.000	14	1.04	65.0
VeChat2	HiCanu	462	91.2	1063634	1063634	0.065	0.000	6	1.09	62.9
CanuCorr2	HiCanu	312	93.9	1557876	1013452	0.089	0.000	30	1.06	63.1
VeChat	HiCanu	423	89.2	1488621	1377622	0.018	0.000	4	1.03	58.2
Canu	HiCanu	429	91.9	790872	457182	0.153	0.000	33	1.06	62.2
-	MetaBooster	226	93.9	1139900	991303	0.060	0.000	44	1.03	61.8
VeChat2	Canu	243	93.0	1077253	603213	0.065	0.000	39	1.02	60.8
CanuCorr2	Canu	320	92.1	736249	414453	0.146	0.000	59	1.05	62.1
VeChat	Canu	283	91.4	787056	483553	0.057	0.000	37	1.03	60.0
-	Canu	336	91.1	670907	382472	0.149	0.000	65	1.05	61.5

Table S4. Assembly performance of the simulated low-complexity dataset using different error corrector combinations. MetaBooster and MetaBooster-HiFi actually use the error corrector combination 'VeChat+Canu error correction'. To demonstrate the power of this combination, we ran 'VeChat+VeChat' (named 'VeChat2') and 'Canu error correction+Canu error correction' (named 'CanuCorr2') for comparison. A single 'HiCanu' was not run because it was designed to only assemble PacBio HiFi reads. The reference size of this low-complexity data is about 63.3 Mbp.

Strain ID	Abundance(%)	Canu	MetaBooster	MetaBooster-HiFi
1053692_0seq	3.1212	1840.28	1125.85	395.45
562971_1seq	3.6807	807.87	692.48	616.58
1172205_1seq	4.7154	5.92	5.43	6.45
267608_0seq	4.8615	228.79	86.84	20.04
402880_0seq	4.9417	877.59	13.33	14.38
1167634_0seq	5.8959	209.01	259.96	198.55
305_16seq	5.9675	356.66	474.63	24.29
305_4seq	7.0385	499.7	348.57	19.47
258594_0seq	7.2311	2.97	0.26	0.58
267377_0seq	7.6586	1889.09	294.84	1.01
652103_0seq	12.0003	0.11	0.28	0.28
1038921_0seq	13.0802	1741.62	13.21	2.53
86192_0seq	19.8074	501	11.38	1.19

Table S5. Error rate per strain of assemblies generated from the simulated low-complexity dataset. The values in 3-5 columns represent the number of errors (mismatches+indels) per 100kbp in the assembly. Rows are ordered by the abundance.

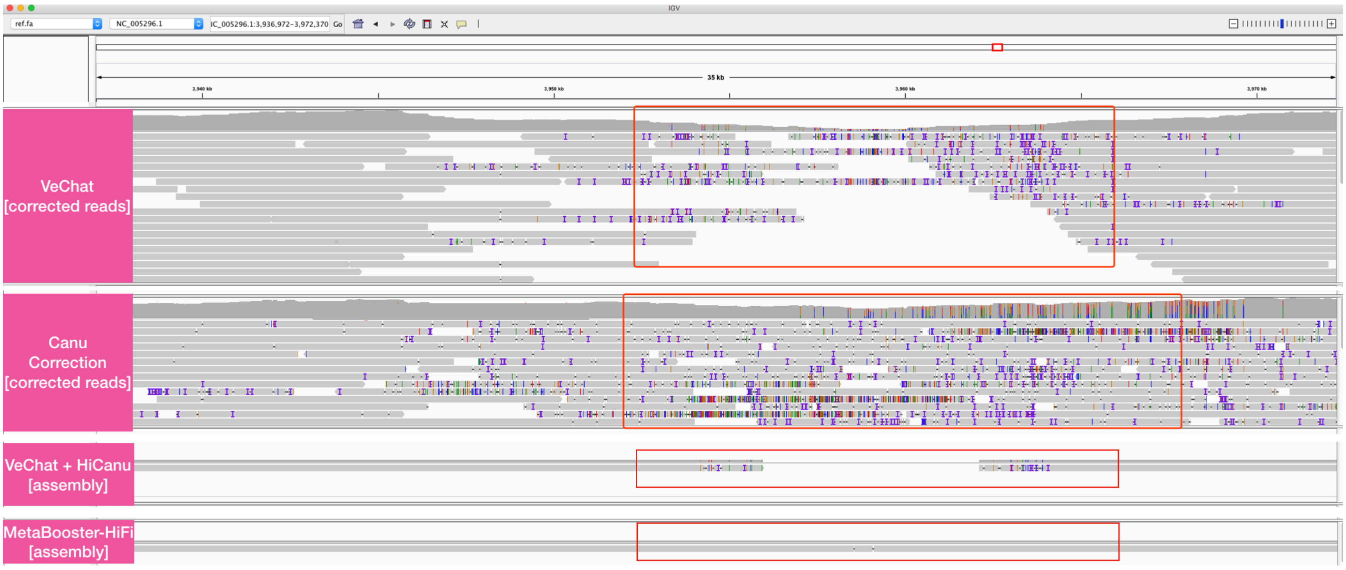

Figure S1. IGV visualization. The red box represents a region showing high sequence divergence (>20%) between different strains in the simulated low-complexity dataset. The top two panels show the alignment of corrected reads(from VeChat or Canu correction) to the reference, whereas the bottom two panels show the alignment of assemblies (from VeChat+HiCanu or MetaBooster-HiFi) to the reference.

2 COMMAND AND VERSION OF TOOLS

- PBSIM2
  `pbsim2 --accuracy-mean 0.9 --hmm_model P6C4.model $ref` # PacBio CLR

- Canu v2.1.1
  #simulated:
  ```
  canu genomeSize=$genomesize rawErrorRate=0.2 -pacbio $raw_read
  ```
  #real:
  ```
  canu genomeSize=$genomesize -pacbio $raw_read
  canu genomeSize=$genomesize -nanopore $raw_read
  ```

  Note that `genomeSize` is set as the corresponding reference size of each dataset, see captions of Table1 and Table2 in the main text for details.

- Flye v2.8.2-b1689
  ```
  flye --meta --nano-raw  $read -t 48
  flye --meta --pacbio-raw  $read -t 48
  ```

- Strainberry v1.1-a90d3b3 ; minimap2 v2.17-r941 ; samtools v1.12
  ```
  minimap2 -ax map-pb -t 48 cns.assembly.fasta $read |
  samtools sort - @$threads > alignment.sorted.bam

  samtools faidx cns.assembly.fasta

  samtools index alignment.sorted.bam

  strainberry -r cns.assembly.fasta -b alignment.sorted.bam -o out -c 48
  ```

- MetaQUAST v5.1.0rc1
  ```
  metaquast.py -r $ref --min-contig 500 -o out --unique-mapping $assembly
  ```

- MetaBooster
  #simulated data (low complexity)
  ```
  metagenome-asm -i reads.fq -g 63m -o out
  -p pb --base -t 48 -m MetaBooster
  ```

  #simulated data (medium complexity)
  ```
  metagenome-asm -i reads.fq -g 374m -o out
  -p pb --base -t 48 -m MetaBooster --split   --split-size 100000
  ```

  #real data (Mock community I: natural whey starter culture data)
  ```
  metagenome-asm -i reads.fq -g 13m -o out -p pb --base -t 48
  -m MetaBooster --split --split-size 70000 --min-identity-cns 0.98
  ```

#real data (Mock community II: Microbial 10-plex data)

```
metagenome-asm -i reads.fq -g 37m -o out -p pb
--base -t 48 -m MetaBooster --split   --split-size 70000
--min-identity-cns 0.98 --scrub
```

#real data (Real human gut microbiome data. Results were generated using the HPC version of VeChat in our manuscript.)

```
metagenome-asm -i reads.fq -g 200m -o out -p ont
--base -t 48 -m MetaBooster --split --split-size 200000
--min-identity-cns 0.98 --scrub
```

- MetaBooster-HiFi
  The commands are the same with those used in MetaBooster, except using `-m MetaBooster-HiFi`
